# ELP6 and PLIN5 Mutations Were Probably Prognostic Biomarkers for Patients With Gastric Cancer

**DOI:** 10.3389/fmed.2022.803617

**Published:** 2022-02-09

**Authors:** Ji Di, Yan Chai, Xin Yang, Haibin Dong, Bo Jiang, Faxiang Ji

**Affiliations:** ^1^Department of Medical Oncology, Affiliated Hospital of Qinghai University, Xining, China; ^2^School of Clinical Medicine, Tsinghua University, Beijing, China; ^3^Department of Gastroenterology, Tsinghua Changgeng Hospital, Tsinghua University, Beijing, China

**Keywords:** gastric cancer, ELP6 mutation, PLIN5 mutation, prognosis, whole exome sequencing

## Abstract

**Purpose:**

Gastric cancer (GC) is the fifth leading cancer around world. And prognosis of patients with GC is still undesirable. Our study aimed to explore potential prognostic biomarkers for patients with GC.

**Methods:**

The clinical samples were collected from the Qinghai University Affiliated Hospital, which were subjected to the whole exome sequencing (WES). The other GC-related data were obtained from The Cancer Genome Atlas (TCGA) database. Cross analyses were done to determine the candidate genes. And the final mutated genes were determined by survival analyses, univariate and multivariate Cox regression analyses. CIBERSORT and GSEA were used for immune cell infiltration analysis and functional enrichment, respectively.

**Results:**

After cross analyses, 160 candidate-mutated genes were identified. And mutated ELP6 and PLIN5 were significantly independently correlated with the overall survival (OS) of patients with GC. Patients with GC with ELP6 and PLIN5 mutations had worse and better prognosis, respectively. Totally 5 types of immune cells were significantly differentially infiltrated in wild-type and mutated ELP6 and PLIN5 GC samples. In mutated ELP6 and PLIN5 GC samples, totally 7 and 11 pathways were significantly enriched, respectively.

**Conclusions:**

The ELP6 and PLIN5 mutations were probably prognostic biomarkers for patients with GC.

## Introduction

Gastric cancer (GC), as the fifth leading cancer and the fourth cause of mortality related to cancer, is a heavy health burden worldwide, leading to ~760,000 deaths per year (1, 2). Meanwhile, the incidence of GC is differential in various areas, and incidence in China and some East Asia countries is relatively high (3). Most patients with GC often show few obvious or specific symptom at an early stage, which results in a lower early detection probability of GC (4). And over 70% patients with GC are diagnosed at an advance stage, whose prognosis is relatively poorer compared with patients diagnosed at early stage, with a median overall survival (OS) of only 10–12 months (5, 6). Despite several risk factors of GC had been documented, such as *Helicobacter pylori* infection (7) and smoking (8), it still remains a great challenge to effectively prevent GC. Currently, a growing number of studies indicated the crucial role of aberrant gene expression in the progression or prognosis of GC (9). For example, PIK3CA mutations have been suggested to be involved in the development of GC and are probably favorable prognostic factor in patients with GC (10). Furthermore, sampling before diagnosis could also be used for predicting survival of patients with GC, which would benefit for a more reliable clinical decision (11). Accordingly, more potential diagnostic or prognostic predictors of GC are still urgently needed to improve the OS of patients with GC.

More recently, with the great development of next-generation sequencing (NGS), the whole exome sequencing (WES) has been also widely applied in many diseases' researches, including cancer (12, 13). WES could help researchers obtain about 85% of known disease-related variants*via* sequencing ~1% of the genome (14), which makes it a powerful tool in cancer researches. And WES has been used for studying the correlation between primary tumors and metastasis in breast cancer (15), colorectal cancer (16), and so on. Moreover, the roles of some gene mutations have also been reported in GC among various population, such as CDH1 (17), TP53 (18), APC (19), ARID1A (20), and so on; however, to the best of our knowledge, the prognostic exploration of GC combining clinical samples' WES with publicly obtained data is limited. Additionally, ELP6 and PLIN5 mutations have been seldom studied in GC samples, but they were studied in some other cancers. The integral Elongator subunit comprising ELP1–ELP6 involves in the cell motility and tumorigenicity (21, 22). And ELP6 (also known as C3ORF75) has been revealed to play an important role in the migration and tumorigenicity of melanoma cells (23). PLIN5 has been reported to be promising prognostic or diagnostic markers in pancreatic cancer and hepatocellular carcinoma (24, 25).

Collectively, in this study, we integrated the WES data of clinical samples, public data in The Cancer Genome Atlas (TCGA) database (https://tcga-data.nci.nih.gov/tcga/), and bioinformatics analyses. Herein, we purposed to explore novel prognostic biomarkers for patients with GC in order to give more alternatives for better clinical strategies.

## Materials and Methods

### Research Objects

All samples used for the WES were collected from the Qinghai University Affiliated Hospital. Totally four groups of participants were included, comprising familial patients with GC (7 patients, case group), healthy relatives of patients with familial GC (4 patients, Kinsfolk group), patients with sporadic GC (3 patients, Sanfa group), and healthy control (2 participants, health group). This study was approved by the Ethics Committee of Affiliated Hospital of Qinghai University (ethic code: P-SL-2018015). And the written informed consents were obtained from all participants.

Moreover, we also downloaded the GC somatic mutation data and RNA-sequencing data from TCGA database. The clinical information of these patients were displayed in Table 1. Furthermore, the GC mRNA expression data in GSE158662 dataset were downloaded from the Gene Expression Omnibus (GEO) database (https://www.ncbi.nlm.nih.gov/geo/), comprising the data of 3 GC specimens and 3 adjacent specimens.

**Table 1:** Clinicopathological characteristics of TCGA patients with gastric carcinoma.

**Characteristics**	**Groups**	**Patients (%)**
Age	Median	67
	Range	30–90
Gender	Female	157 (35.93%)
	Male	280 (64.07%)
TNM stage	I	57 (13.04%)
	II	130 (29.75%)
	III	181 (41.42%)
	IV	43 (9.84%)
	Unknown	26 (5.95%)
Invasion depth	T1	23 (5.26%)
	T2	91 (20.82%)
	T3	197 (45.08%)
	T4	117 (26.77%)
	TX	9 (2.06%)
Lymph node metastasis	N0	130 (29.75%)
	N1	117 (26.77%)
	N2	84 (19.22%)
	N3	87 (19.91%)
	NX	17 (3.89%)
	Unknown	2 (0.46%)
Distant metastasis	M0	385 (88.10%)
	M1	30 (6.86%)
	MX	22 (5.03%)
Grade	G1	12 (2.75%)
	G2	159 (36.38%)
	G3	257 (58.81%)
	GX	9 (2.06%)
ELP6 mutation	Wild	432 (98.86%)
	Mutation	5 (1.14%)
PLIN mutation	Wild	423 (96.80%)
	Mutation	14 (3.20%)
Status	Alive	240 (54.92%)
	Dead	169 (38.67%)
	Unknown	28 (6.41%)

*TCGA, The Cancer Genome Atlas*.

### Whole Exome Sequencing

The Agilent SureSelect Human All Exon V6 Kit (Agilent, Santa Clara, CA, USA) was used to construct and capture the DNA of exon regions of all sample, which were then sequenced on the Illumina platform. All reads were aligned with the human reference genome (hg38) utilizing the Burrows-Wheeler-Alignment (BWA) Tool (Wellcome Trust Sanger Institute, Cambridge, UK) (26) software, and the Sentieon Haplotyper module of BWA was used for SNV and Indel detecting. Then the detected SNP and Indel were annotated in the ANNOVAR (University of Pennsylvania, Philadelphia, PA, USA) software (27).

### Candidate Gene Screening

The mutation data obtained from the WES were firstly screened, and the synonymous mutation and the sites located in the intergenic and intron regions were removed. Compared with health group, the genes only mutated in case group were selected. Compared with the Kinsfolk group, the genes only mutated in the case group were selected. Compared with the Sanfa group, the genes only mutated in the case group were selected. And the cross analyses of these three pair comparison finally determined potential candidate genes.

### Survival Analyses

The correlation between gene mutations and OS was analyzed according to the Kaplan–Meier (KM) method. And the mutant genes related to the prognosis of GC were then screened basing on the univariate Cox regression analysis, utilizing survival and survminer package of R. And the multivariate Cox regression analysis was adopted to determine whether prognosis-related mutant genes were independent prognostic factors.

### Immune Cell Infiltration

The relative proportions of 22 kinds of immune cells in GC samples downloaded from the TCGA database were analyzed in the software CIBERSORT (Alizadeh Lab and Newman Lab, Stanford, CA, USA) (28). Based on the gene expression matrix, the CIBERSORT method could characterize the immune cell composition in each sample according to the deconvolution algorithm.

### Gene Set Enrichment Analysis

The Gene Set Enrichment Analysis (GSEA) was performed on the mutant and wild-type GC samples, on the basis of the Kyoto Encyclopedia of Genes and Genomes (KEGG) pathway gene sets (Version 7.4) under Molecular Signatures Database (MSigDB) (https://www.gsea-msigdb.org/gsea/index.jsp), using the GSEA software (Version 4.0.3) (29). And the number of gene set permutations was 1,000 during the analysis.

### Statistical Analyses

The immune cell infiltration difference among various groups was compared using the Wilcoxon signed-rank test. The significant threshold was *p* < 0.05. All statistical analyses were done in R (version v3.6.1).

## Results

### The Identification of Candidate Genes

**Figure 1:**
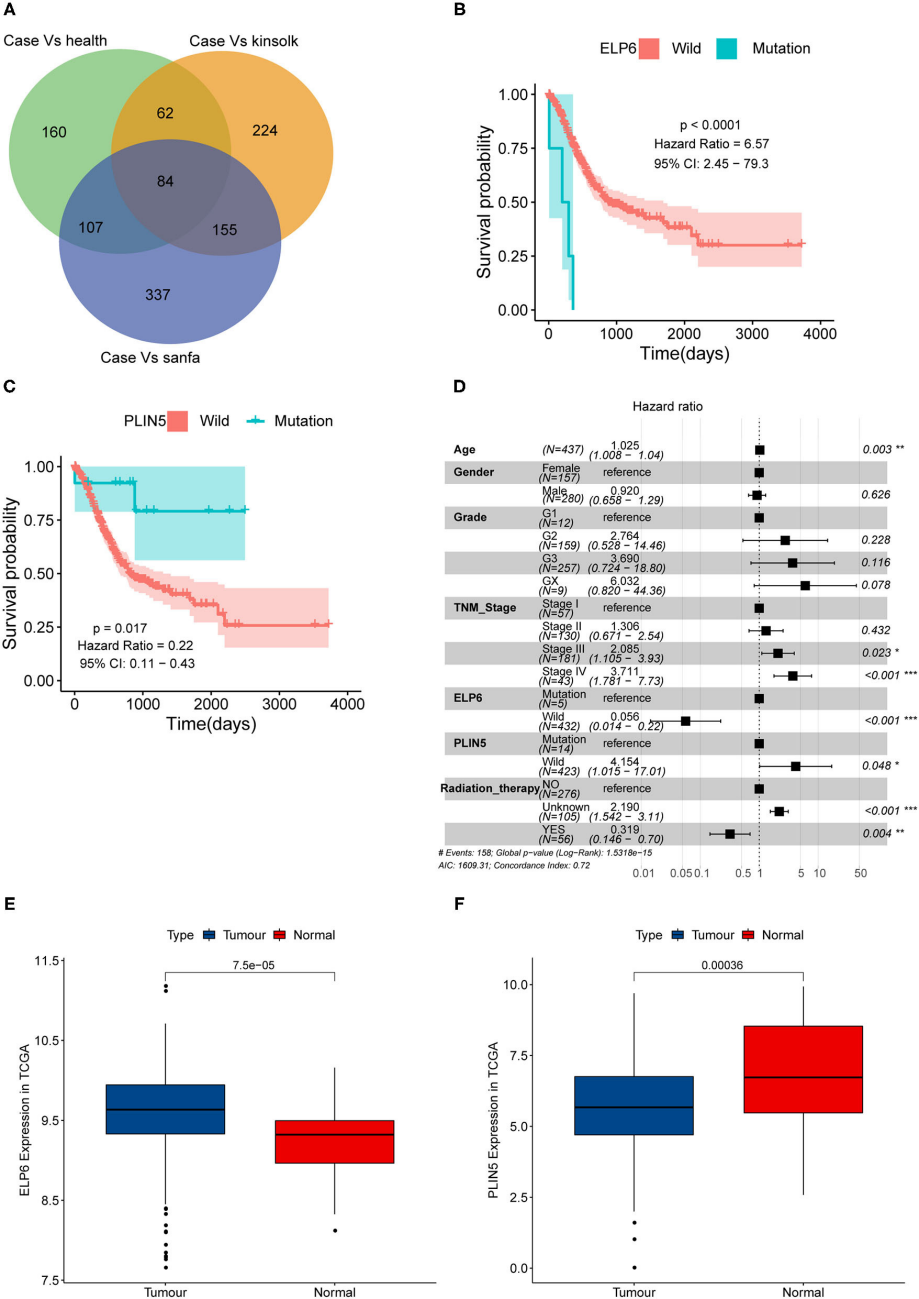
ELP6 and PLIN5 mutations were related to the prognosis of patients with GC. **(A)** Venn diagram of screening candidate mutated genes. **(B)** KM survival curve showed that ELP6 mutation was a poor prognostic indicator. **(C)** PLIN5 mutation was a good prognostic indicator for patients with GC. **(D)** Multivariate Cox regression analysis results. Hazard ratio (HR) > 1, samples have a higher risk of death; and HR < 1, samples have a lower death risk. **(E,F)** The expression of ELP6 and PLIN5 in GC samples and adjacent samples, respectively. GC, gastric cancer; KM, Kaplan–Meier. * *p*-value < 0.05; ** *p*-value < 0.01; *** p-value < 0.001.

First, we screened the potential candidate genes based on the results of WES. Compared with the health group, totally 413 genes mutated only in the case group while did not mutate in the health group. Compared with the Kinsfolk group, 525 genes mutated only in the case group. Compared with the Sanfa group, 683 genes mutated only in the case group. After cross analysis, the results were displayed in Venn diagram (Figure 1A). Then the 160 mutated genes found in the case vs. health groups were potential candidate genes (green part in Figure 1A). And the detailed mutated genes and candidate genes were listed in Supplementary Table 1.

### ELP6 and PLIN5 Mutations Were Independent Prognostic Factors for GC

The somatic mutation data in the TCGA database was divided into wild-type and mutation groups according to whether the candidate genes mutated or not. And the univariate Cox regression analysis was conducted on the wild-type and mutant samples. We found that mutated ELP6 and PLIN5 were significantly related to the OS of patients with GC (*p* < 0.05). Moreover, the OS of mutated ELP6 GC samples was undesirable (HR = 6.57, 95%CI: 2.45–79.3, *p* < 0.0001) (Figure 1B), but mutated PLIN5 GC samples had a better prognosis (HR = 0.22, 95%CI: 0.11–0.43, *p* = 0.017) (Figure 1C).

Furthermore, a multivariate Cox regression analysis included 7 factors was performed to determine whether ELP6 and PLIN5 were independent prognostic indicators, comprising age, gender, TNM stage, grade, radiation therapy, ELP6, and PLIN5. The results suggested that ELP6 was still significantly associated with the OS of patients with GC, and taking wild-type as reference, GC samples with ELP6 mutation had a higher risk of death (HR = 15.970, 95%CI: 4.222–60.406, *p* < 0.001) (Figure 1D). Furthermore, PLIN5 was also significantly correlated with the OS of patients with GC, and when compared with the wild-type reference, mutated PLIN5 was a good prognostic factor for patients with GC (HR = 0.175, 95%CI: 0.043–0.715, *p* = 0.0152) (Figure 1D). Both ELP6 and PLIN5 were independent prognostic indicators; furthermore, age, TNM stage, and radiation therapy were also independent prognostic factors for patients with GC. Moreover, the expression of ELP6 and PLIN5 was compared between GC and adjacent samples. We found that ELP6 was significantly highly expressed in GC samples (Figure 1E), whereas PLIN5 showed significantly higher expression in adjacent samples (Figure 1F). In GSE158662 dataset, ELP6 was also highly expressed in GC samples (Supplementary Figure 1A), and PLIN5 showed significantly lower expression in GC samples (Supplementary Figure 1B).

### ELP6 and PLIN5 Mutations Were Related to the Differential Immune Cell Infiltration in GC Samples

**Figure 2:**
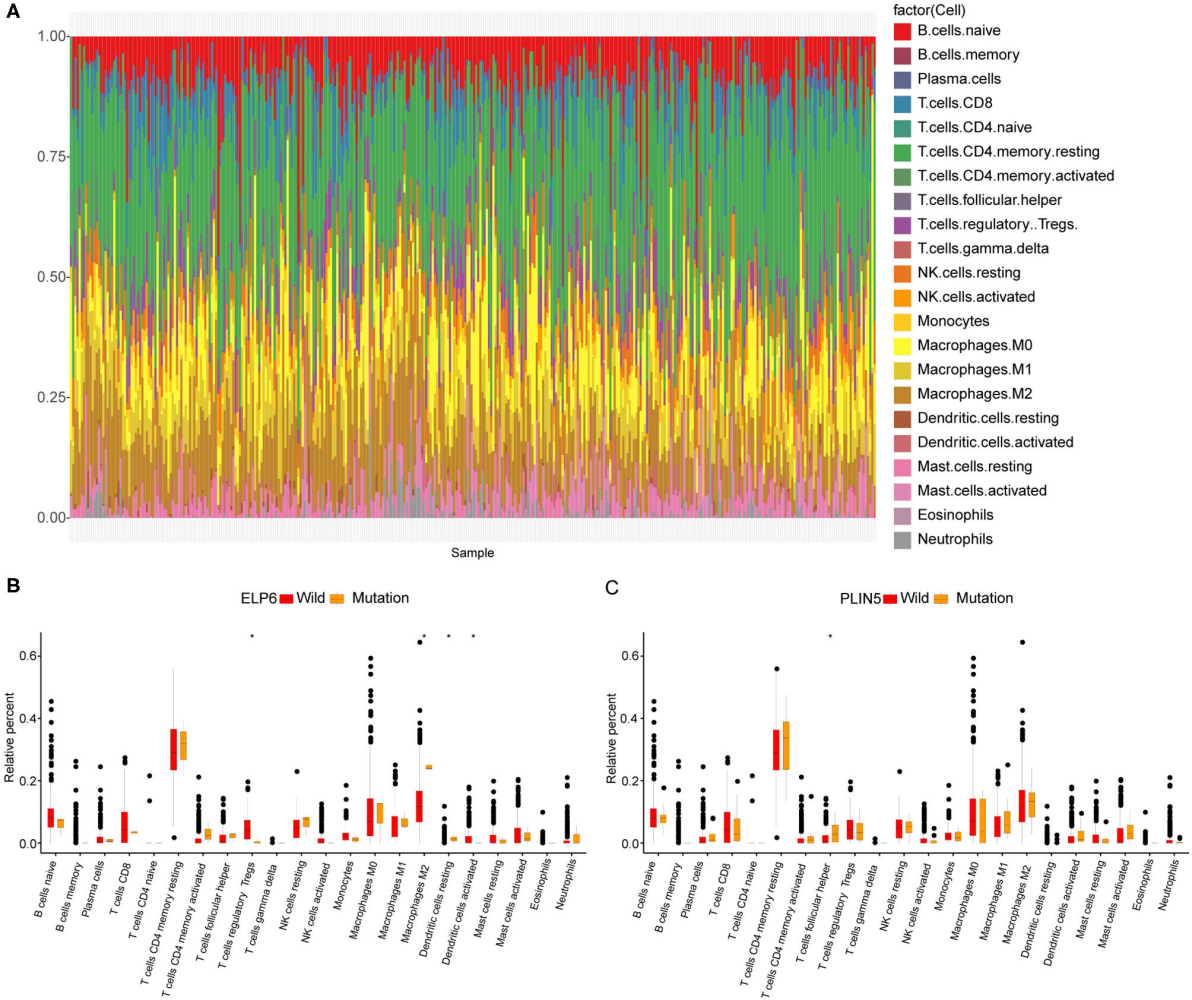
Immune cell infiltration in wild-type and mutant patients with gastric cancer (GC). **(A)** The infiltration proportions of 22 types of immune cells in all patients with GC. **(B)** Tumor infiltrating immune cell difference between wild-type and mutated ELP6 GC samples. **(C)** Tumor infiltrating immune cell difference between wild-type and mutated PLIN5 GC samples. *p-value < 0.05.

Subsequently, we also explored the immune cell infiltration in all GC samples in TCGA. The results of analysis in CIBERSORT indicated that there were differential infiltrating proportions of 22 types of immune cells in various GC samples (Figure 2A). Additionally, four kinds of immune cells were significantly differentially infiltrated between wild-type and mutated ELP6 GC samples, including regulatory T cells (Tregs), M2 Macrophages, dendritic cells resting, and dendritic cells activated (*p* < 0.05) (Figure 2B). Between wild-type and mutated PLIN5 GC samples, T follicular helper cells showed significantly differential infiltration (*p* < 0.05) (Figure 2C). These significantly differentially infiltrated immune cells probably contributed to the differential prognoses of wild-type and mutant patients with GC.

### ELP6- and PLIN5-Related Pathways Revealed Through GSEA

**Figure 3:**
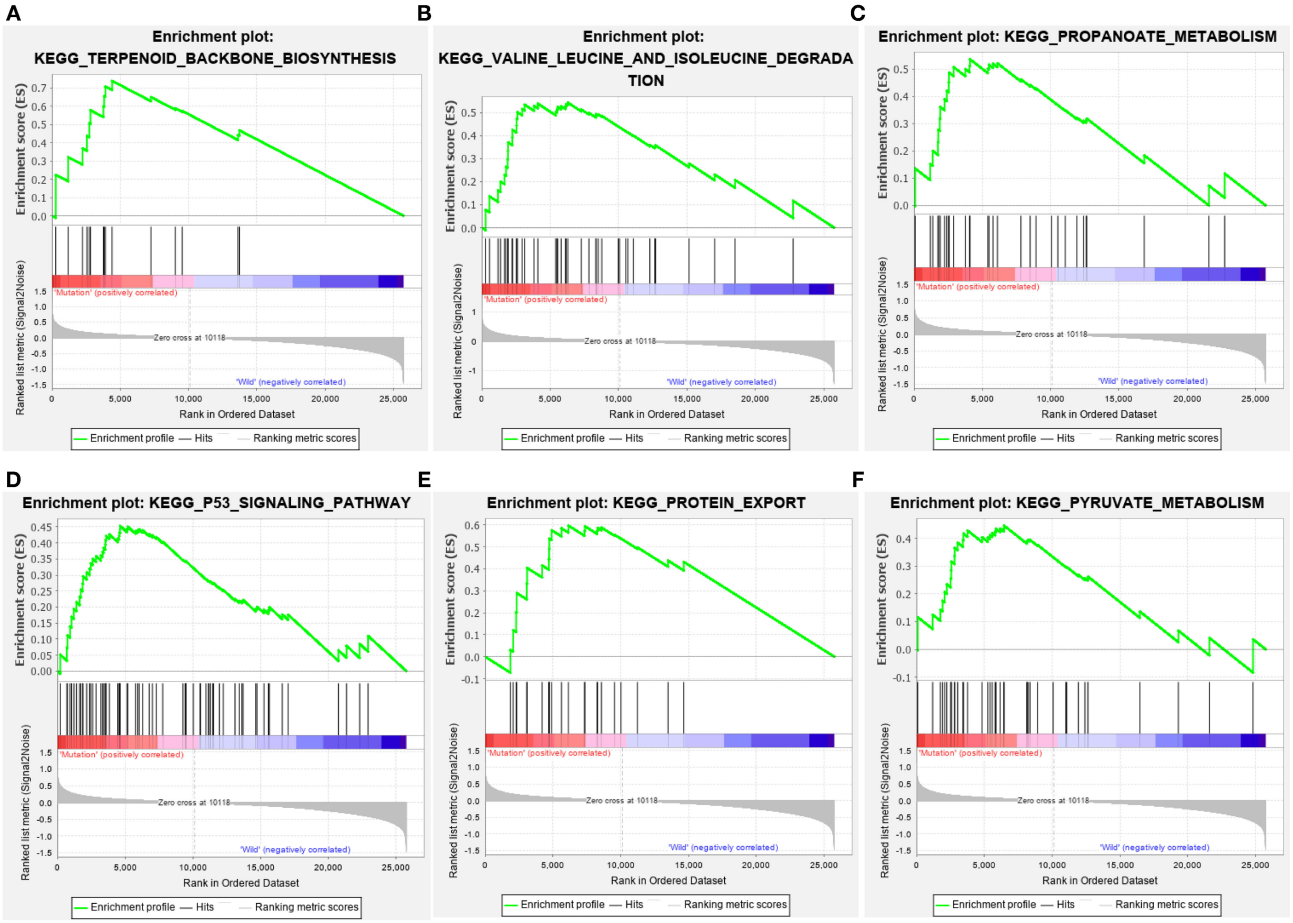
The significantly enriched pathways in mutated ELP6 GC samples based on GSEA. **(A)** Terpenoid backbone biosynthesis pathway, **(B)** valine leucine and isoleucine degradation pathway, **(C)** propanoate metabolism pathway, **(D)** p53 signaling pathway, **(E)** protein export pathway, and **(F)** pyruvate metabolism pathway. GC, gastric cancer; GSEA, Gene Set Enrichment Analysis.

**Figure 4:**
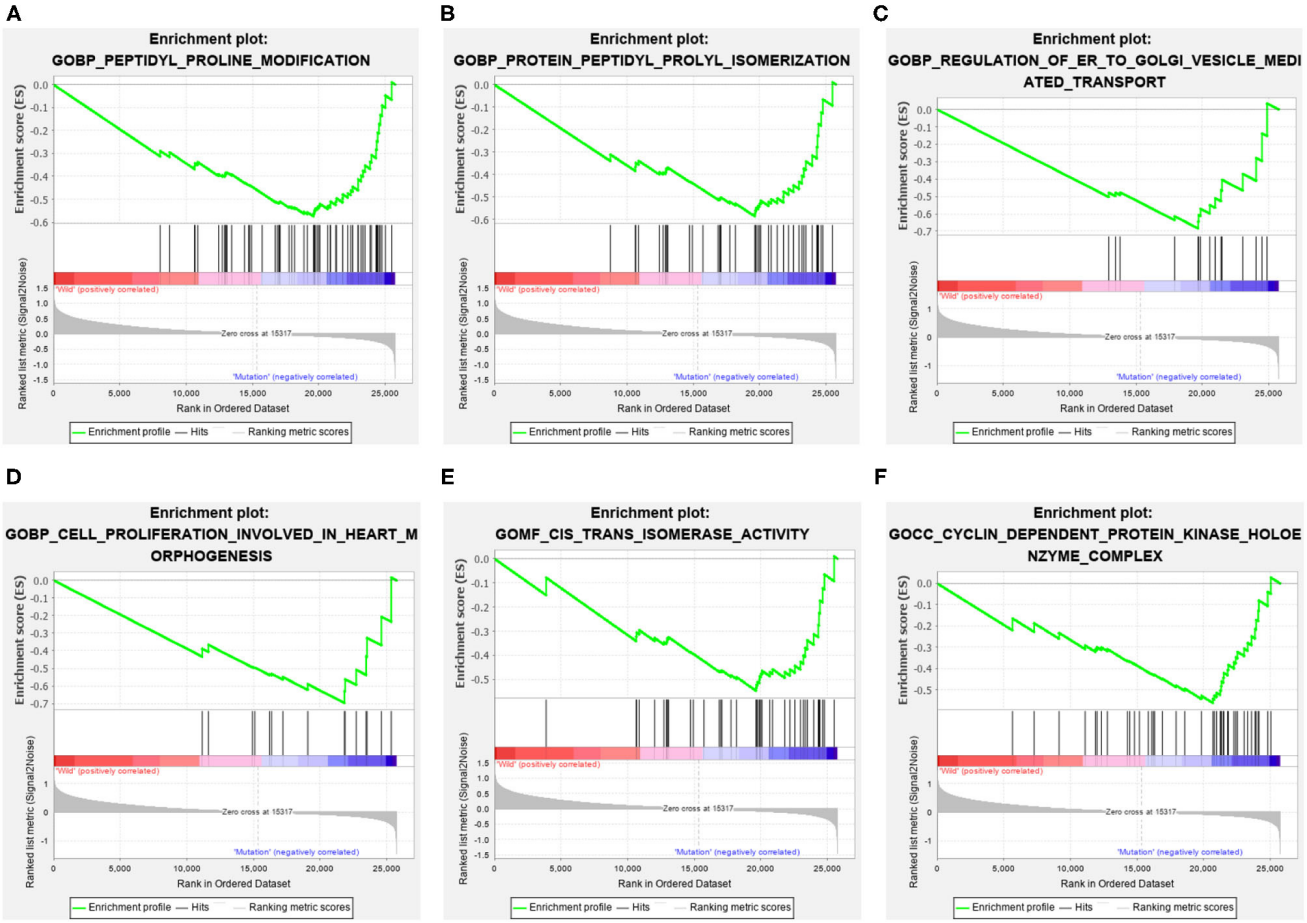
The significantly enriched GO terms in mutated ELP6 GC samples based on GSEA. **(A)** Peptidyl proline modification, **(B)** protein peptidyl prolyl isomerization, **(C)** regulation of ER to golgi vesicle-mediated transport, **(D)** cell proliferation involved in heart morphogenesis, **(E)** cis trans isomerase activity, and **(F)** cyclin-dependent protein kinase holoenzyme complex. GO, Gene Ontology; GC, gastric cancer; GSEA, Gene Set Enrichment Analysis.

**Figure 5:**
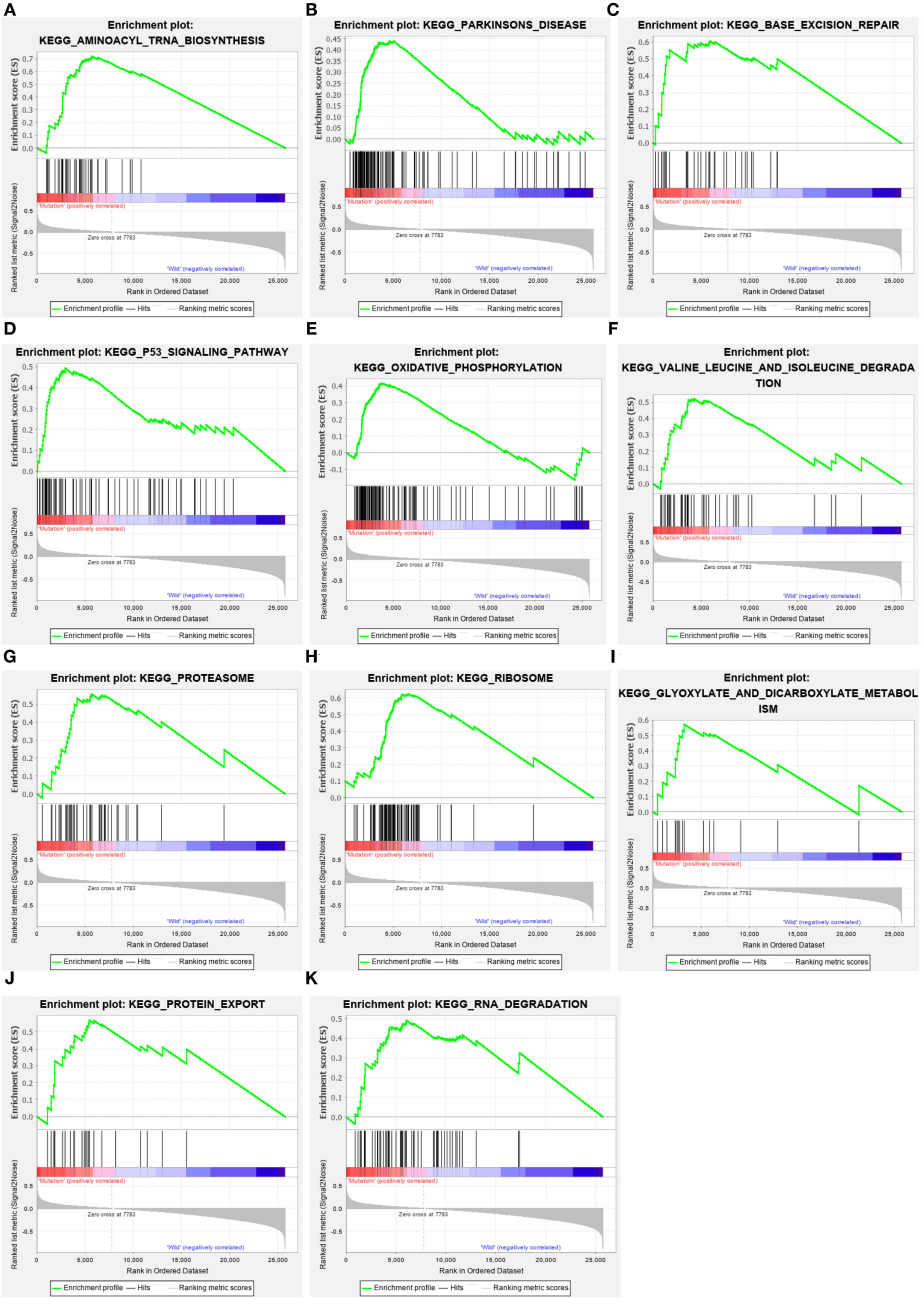
The significantly enriched pathways in mutated PLIN5 GC samples based on GSEA. **(A)** Aminoacyl-tRNA biosynthesis pathway, **(B)** Parkinson's disease pathway, **(C)** base excision repair pathway, **(D)** p53 signaling pathway, **(E)** oxidative phosphorylation pathway, **(F)** valine leucine and isoleucine degradation pathway, **(G)** proteasome pathway, **(H)** ribosome pathway, **(I)** glyoxylate and dicarboxylate metabolism pathway, **(J)** protein export pathway, and **(K)** RNA degradation pathway. GC, gastric cancer; GSEA, Gene Set Enrichment Analysis.

**Figure 6:**
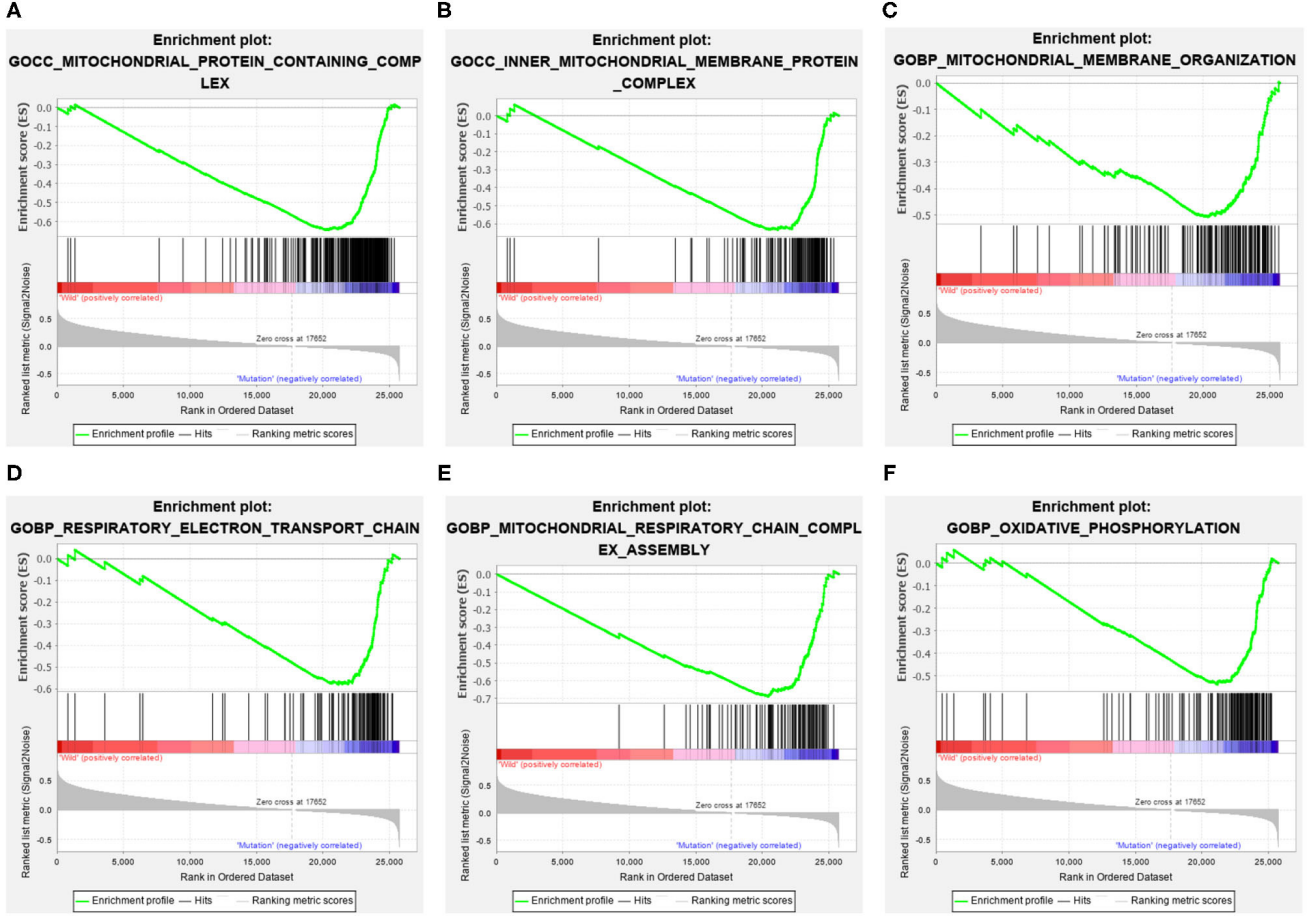
The significantly enriched GO terms in mutated PLIN5 GC samples based on GSEA. **(A)** Mitochondrial protein containing complex, **(B)** inner mitochondrial membrane protein complex, **(C)** mitochondrial membrane organization, **(D)** respiratory electron transport chain, **(E)** mitochondrial respiratory chain complex assembly, and **(F)** oxidative phosphorylation. GO, Gene Ontology; GC, gastric cancer; GSEA, Gene Set Enrichment Analysis.

The GC samples in TCGA database were then subjected to the GSEA to obtain the mutated ELP6- and PLIN5-related functional information. Our results showed that in mutated ELP6 GC samples, terpenoid backbone biosynthesis pathway, valine leucine and isoleucine degradation pathway, propanoate metabolism pathway, p53 signaling pathway, protein export pathway, pyruvate metabolism signaling pathways (Figure 3; Table 2), and 56 Gene Ontology (GO) terms (Figure 4; Supplementary Table 2) were significantly enriched. And in mutated PLIN5 GC samples, aminoacyl-tRNA biosynthesis pathway, Parkinson's disease pathway, base excision repair pathway, p53 signaling pathway, oxidative phosphorylation pathway, valine leucine and isoleucine degradation pathway, proteasome pathway, ribosome pathway, glyoxylate and dicarboxylate metabolism pathway, protein export pathway, RNA degradation pathway (Figure 5; Table 3), and 176 GO terms (Figure 6; Supplementary Table 2) were significantly enriched. These functional pathways indicated the potential role of mutated ELP6 and PLIN5 in the progression and prognosis of GC samples.

**Table 2:** GSEA-enriched KEGG pathways in ELP6 wild and ELP6 mutation.

**Name**	**SIZE**	**NES**	**NOM *p*-value**
**ELP6 wild**			
KEGG_VASOPRESSIN_REGULATED_WATER_REABSORPTION	44	−1.6403573	0.03112
KEGG_PHENYLALANINE_METABOLISM	18	−1.5563954	0.0316
KEGG_TYPE_II_DIABETES_MELLITUS	47	−1.5366147	0.0289
KEGG_TIGHT_JUNCTION	132	−1.467734	0.0365
**ELP6 mutation**			
KEGG_TERPENOID_BACKBONE_BIOSYNTHESIS	15	1.8711467	0.0037
KEGG_VALINE_LEUCINE_AND_ISOLEUCINE_DEGRADATION	44	1.7817888	0.0162
KEGG_PROPANOATE_METABOLISM	32	1.7669461	0.0056
KEGG_P53_SIGNALING_PATHWAY	68	1.7138146	0.0150
KEGG_PROTEIN_EXPORT	23	1.6627951	0.0138
KEGG_PYRUVATE_METABOLISM	40	1.576139	0.0407

*GSEA, Gene Set Enrichment Analysis; KEGG, Kyoto Encyclopedia of Genes and Genomes*.

**Table 3:** GSEA-enriched KEGG pathways in PLIN5 wild and PLIN5 mutation.

**Name**	**SIZE**	**NES**	**NOM *p*-value**
**PLIN5 wild**			
KEGG_TIGHT_JUNCTION	132	−1.5699017	0.0305
KEGG_ABC_TRANSPORTERS	44	−1.5161897	0.0412
KEGG_NEUROACTIVE_LIGAND_RECEPTOR_INTERACTION	271	−1.4389659	0.0133
**PLIN5 mutation**			
KEGG_AMINOACYL_TRNA_BIOSYNTHESIS	41	2.0124779	0
KEGG_PARKINSON'S_DISEASE	113	1.8553538	0.0227
KEGG_BASE_EXCISION_REPAIR	33	1.8451898	0.0094
KEGG_P53_SIGNALING_PATHWAY	68	1.8282967	0.0115
KEGG_OXIDATIVE_PHOSPHORYLATION	116	1.74625	0.0468
KEGG_VALINE_LEUCINE_AND_ISOLEUCINE_DEGRADATION	44	1.735111	0.0308
KEGG_PROTEASOME	44	1.7183533	0.02148
KEGG_RIBOSOME	87	1.7036082	0.0468
KEGG_GLYOXYLATE_AND_DICARBOXYLATE_METABOLISM	16	1.646111	0.0306
KEGG_PROTEIN_EXPORT	23	1.6215007	0.0200
KEGG_RNA_DEGRADATION	57	1.615687	0.04340

## Discussion

In our present study, through the comprehensive analyses of the WES data of locally clinical GC samples and public GC data in TCGA database, mutated ELP6 and PLIN5 were found to be promising prognostic markers for patients with GC. Furthermore, ELP6 and PLIN5 mutations were both correlated with the differential immune cell infiltration in GC samples, which contributed to the differential prognoses of patients with GC.

The onset and progression of GC is usually affected by many factors, such as genetic factors (30), behavioral factors (31), race (32), and so on. Accordingly, based on the results of WES, we conducted multiple cross analyses to avoid genetic or environmental influences and to determine the candidate mutated genes. Totally 160 mutated genes were identified. Subsequently, univariate and multivariate Cox regression analyses and GC somatic mutation data in TCGA together determined that mutated ELP6 and PLIN5 were independent prognostic predictors of GC. And patients with GC with ELP6 and PLIN5 mutations had relatively poor and good OS, respectively. ELP6 encodes a crucial subunit of the Elongator acetyltransferase complex, and Elongator influences the transcript elongation and protein translation (22). Some of the ELP members showed differential roles in various cancers, for example, aberrant expression of Elp3 or Elp4 promoted the migration and invasion of hepatocellular carcinoma cells (22). In mouse model, Elongator mutation has been reported to induce the neurodegeneration and ataxia-like behavior (33). Moreover, ELP6 has been evidenced to play an important role in migration and tumorigenicity of melanoma cells (23), which reminded us that mutated ELP6 had a similar function in GC and thus was related to the poor prognosis of GC. PLIN5 (perilipin 5) could coat intracellular lipid storage droplets and protect them from lipolytic degradation. And as one of the redox-dependent factors of tumor, PLIN5 indirectly affected the redox state of adipose tissue in GC (34), which was potentially related to the prognosis of patients with GC. Additionally, the prognostic value of PLIN5 has been explored in many cancers, including breast cancer (35), pancreatic cancer (24), hepatocellular carcinoma (25), and so on; however, we first investigated the prognostic role of mutated PLIN5 in patients with GC. All the above evidence indicated that ELP6 and PLIN5 mutations were probably prognostic biomarkers for patients with GC.

Complex immune responses of the host have been the vital part of studying GC and its immunotherapy (36). Thus, we also studied the immune cell infiltration, and totally 5 types of immune cells were significantly differentially infiltrated in wild-type and mutated ELP6 and PLIN5 GC samples. Tregs played a key role in the development and progression of GC; furthermore, some subsets of Tregs in GC microenvironment induced the immunosuppression especially (37–39), implying its possible influence on mutated ELP6 and PLIN5 samples. Moreover, M2 Macrophages contributed to the progression and metastasis of GC (40, 41), which potentially affected the prognosis of patients with GC. The dysregulation of T follicular helper cells has been documented to involve in the inflammation and tumor development in GC, thus was correlated with the worse outcomes of patients (42), which was consistent with our findings. Collectively, considering the complex interactions between immune cells and GC, our findings deserved further exploration in the near future.

Additionally, more functional information of mutated ELP6 and PLIN5 in GC samples was obtained*via* GSEA. In mutated ELP6 and PLIN5 GC samples, totally 7 and 11 pathways were significantly enriched, respectively. We noticed that the P53 signaling pathway was also significantly enriched, which was regulated by multiple factors in various tumors and also was demonstrated to regulate GC cell function indirectly (43, 44). In addition, some metabolism-related pathways were also significantly enriched, such as propanoate metabolism pathway, pyruvate metabolism pathway, valine leucine and isoleucine degradation pathway, and so on. Our results provided more fundamental information of the role of ELP6 and PLIN5 mutations in GC samples, which deserved further investigated.

## Conclusions

To summarize, we firstly revealed the prognostic value of mutated ELP6 and PLIN5 in GC*via* integrating the WES data of local GC samples and publicly obtained data. The ELP6 mutation is an independent poor prognostic marker for patients with GC, but patients with GC with mutated PLIN5 had relatively good prognoses.

## Data Availability Statement

The data presented in the study are deposited in the National Center for Biotechnology Information repository, accession number PRJNA791478.

## Ethics Statement

The studies involving human participants were reviewed and approved by Ethics Committee of Affiliated Hospital of Qinghai University. The patients/participants provided their written informed consent to participate in this study.

## Author Contributions

JD, BJ, and FJ designed the study and drafted the manuscript. JD and YC performed the data curation and analysis. XY and HD analyzed and interpreted the results. All authors reviewed the manuscript and approved the final manuscript to be published.

## Funding

This research was funded by the Technology Department of Qinghai province (Grant Number: 2018-HZ-814 and 2018-ZJ-702).

## Conflict of Interest

The authors declare that the research was conducted in the absence of any commercial or financial relationships that could be construed as a potential conflict of interest.

## Publisher's Note

All claims expressed in this article are solely those of the authors and do not necessarily represent those of their affiliated organizations, or those of the publisher, the editors and the reviewers. Any product that may be evaluated in this article, or claim that may be made by its manufacturer, is not guaranteed or endorsed by the publisher.

## Supplementary Material

Supplementary Figure 1 The expression of ELP6 and PLIN5 in GSE158662 dataset. **(A,B)** The expression of ELP6 and PLIN5 in GC samples and adjacent samples, respectively. GC, gastric cancer.

Supplementary Table 1 Mutant genes in each group.

Supplementary Table 2 Detailed results of GSEA.
