# Supplementary figures and images for: ELP6 and PLIN5 Mutations Were Probably Prognostic Biomarkers for Patients With Gastric Cancer
Source: Front Med (Lausanne). 2022 Feb 9;9:803617. doi: 10.3389/fmed.2022.803617 (PMC8864479; doi:10.3389/fmed.2022.803617)

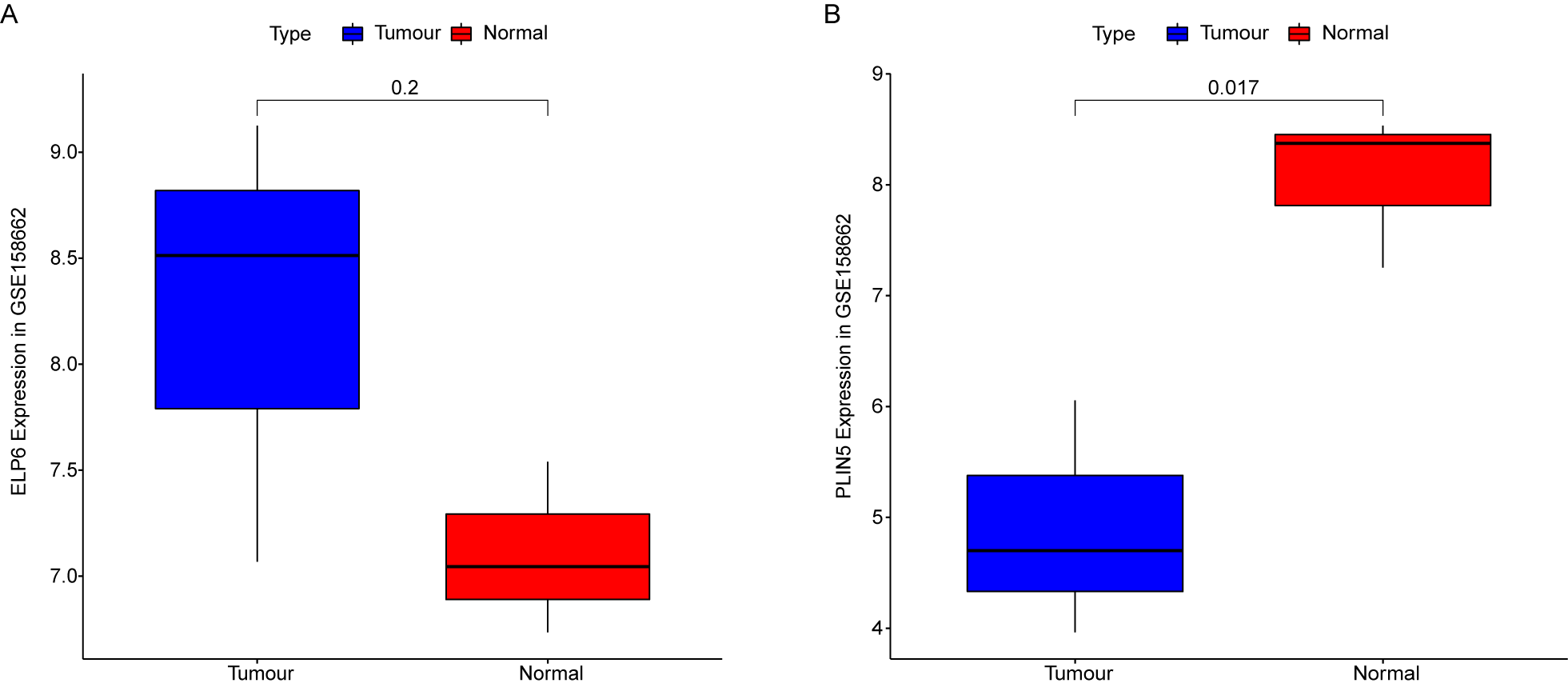

Supplement: Supplementary Figure 1 — The expression of ELP6 and PLIN5 in GSE158662 dataset. (A,B) The expression of ELP6 and PLIN5 in GC samples and adjacent samples, respectively. GC, gastric cancer. [file Image_1.TIF]
